# Nrf2 Activation Attenuates Chronic Constriction Injury-Induced Neuropathic Pain via Induction of PGC-1*α*-Mediated Mitochondrial Biogenesis in the Spinal Cord

**DOI:** 10.1155/2021/9577874

**Published:** 2021-10-21

**Authors:** Jia Sun, Jia-Yan Li, Long-Qing Zhang, Dan-Yang Li, Jia-Yi Wu, Shao-Jie Gao, Dai-Qiang Liu, Ya-Qun Zhou, Wei Mei

**Affiliations:** ^1^Anesthesiology Institute, Tongji Hospital, Tongji Medical College, Huazhong University of Science and Technology, Wuhan, China; ^2^Department of Anesthesiology and Pain Medicine, Tongji Hospital, Tongji Medical College, Huazhong University of Science and Technology, Wuhan, China

## Abstract

**Background:**

Neuropathic pain is a debilitating disease with few effective treatments. Emerging evidence indicates the involvement of mitochondrial dysfunction and oxidative stress in neuropathic pain. Nuclear factor erythroid 2-related factor 2 (Nrf2) is a potent regulator of the antioxidant response system. In this study, we investigated whether RTA-408 (RTA, a novel synthetic triterpenoid under clinical investigation) could activate Nrf2 and promote mitochondrial biogenesis (MB) to reverse neuropathic pain and the underlying mechanisms.

**Methods:**

Neuropathic pain was induced by chronic constriction injury (CCI) of the sciatic nerve. Pain behaviors were measured via the von Frey test and Hargreaves plantar test. The L4-6 spinal cord was collected to examine the activation of Nrf2 and MB.

**Results:**

RTA-408 treatment significantly reversed mechanical allodynia and thermal hyperalgesia in CCI mice in a dose-dependent manner. Furthermore, RTA-408 increased the activity of Nrf2 and significantly restored MB that was impaired in CCI mice in an Nrf2-dependent manner. Peroxisome proliferator-activated receptor-gamma coactivator-1alpha (PGC-1*α*) is the key regulator of MB. We found that the PGC-1*α* activator also induced a potent analgesic effect in CCI mice. Moreover, the antinociceptive effect of RTA-408 was reversed by the preinjection of the PGC-1*α* inhibitor.

**Conclusions:**

Nrf2 activation attenuates chronic constriction injury-induced neuropathic pain via induction of PGC-1*α*-mediated mitochondrial biogenesis in the spinal cord. Our results indicate that Nrf2 may be a potential therapeutic strategy to ameliorate neuropathic pain and many other disorders with oxidative stress and mitochondrial dysfunction.

## 1. Introduction

Neuropathic pain arises due to a primary lesion or dysfunction affecting the somatosensory nervous system, which markedly impairs the patients' quality of life and reduces individual productivity [1, 2]. Unfortunately, the efficacy of pharmacologic treatment is limited and is associated with side effects and risks of abuse [3, 4]. Therefore, identification of novel therapeutic strategies is considered to be a significant and unmet need.

Despite rapid advance over the past decades, the mechanisms underlying the development and maintenance of chronic pain remain to be elucidated [5–7]. However, accumulating evidence indicates that oxidative stress and mitochondrial dysfunction are involved in various animal models of chronic pain [8–11]. Mitochondrial biogenesis (MB) is the process of producing new functional mitochondria, which could restore mitochondrial function after various stimuli or injuries [12–15]. The main regulatory factor of MB is peroxisome proliferator-activated receptor coactivator 1*α* (PGC-1*α*). PGC-1*α* displays its functions by increasing many transcription factors, including nuclear respiratory factors 1 and 2 (NRF1 and NRF2). NRF1 and PGC-1*α* coactivate the transcriptional function of mitochondrial transcription factor A (TFAM) that directly promotes transcription and replication of the mitochondrial genome [16]. The antioxidant response element (ARE) is a DNA regulatory element, mainly binding with nuclear factor erythroid 2-related factor 2 (Nrf2) to activate these genes (such as heme oxygenase-1 (HO-1), NRF1, and Hmox1). Under physiologic conditions, Nrf2 is sequestered to the cytoplasm by Kelch-like ECH-associated protein 1- (Keap1-) Nrf2 complex and ubiquitin degradation. However, oxidative stress triggers the dissociation of the Keap1-Nrf2 complex. Nrf2 then enters the nucleus and activates ARE to regulate the transcription of antioxidant-related genes [17–19]. Nrf2 is believed to be a master regulator of endogenous antioxidant defense and MB [20–22]. The principal role of Nrf2/ARE in MB has been revealed by HO-1 activity in several models [20, 22]. Our previous study demonstrated that oltipraz and rosiglitazone significantly attenuate paclitaxel-induced neuropathic pain via induction of the Nrf2/HO-1 signaling pathway in the spinal cord [23, 24]. Moreover, several studies illustrated that Nrf2 promoted MB via the regulation of PGC-1*α* [25, 26]. Thus, we suggested that Nrf2 may be an attractive therapeutic target to stimulate MB under neuropathic pain conditions in a PGC-1*α* dependent manner.

RTA-408 (RTA), a novel Nrf2 activator, is a member of the synthetic oleanane triterpenoid compounds [27–30]. It is currently being used in several clinical trials for the prevention and treatment of a variety of diseases, including radiation-induced dermatitis (NCT02142959) [31], advanced solid cancers (melanoma or non-small-cell lung cancer) (NCT02029729) [32], and Friedreich ataxia (NCT02255435) [33]. A previous study has shown that RTA-408 plays a critical role in mitigating radiation-induced bone marrow suppression by activating Nrf2 [34]. Notably, a recent study has demonstrated that RTA-408 could stimulate MB and benefit patients with mitochondrial myopathy (NCT02255422) [35]. Another study demonstrated that activation of Nrf2 with RTA-408 exerted a neuroprotective and disease-modifying effect by inhibiting reactive oxygen species production, mitochondrial depolarization, and neuronal death [36]. Thus, this study investigated the promising effect of RTA-408 in the CCI model, and we hypothesized that RTA-408 may exert analgesic effects via induction of PGC-1*α*-mediated MB in the spinal cord in a mice model of chronic constriction injury (CCI).

## 2. Methods

### 2.1. Animals

Adult male C57BL/6J mice weighing 23-25 g from Tongji Medical College, Wuhan, China, were used. All mice were housed in a temperature- and humidity-controlled environment, under a 12 : 12 light-dark cycle, and with free access to a standard diet and tap water ad libitum. All behavioral tests were performed from 8:00 A.M. to 4:00 P.M. All experimental procedures were performed following the approval of the ethics committee of the Animal Care and Use Committee of Huazhong University of Science and Technology.

### 2.2. Drug Administration

RTA-408, purchased from Selleck (Houston, TX, USA), was dissolved in 10% DMSO and 10% Tween 80 in sterile saline. The Nrf2 inhibitor, trigonelline hydrochloride (Trig), purchased from Selleck (Houston, TX, USA), was dissolved in sterile saline. A PGC-1*α* activator, ZLN005 (ZLN), purchased from MedChemExpress (Monmouth Junction, NJ, USA), was dissolved in 10% DMSO and 10% Tween 80 in sterile saline. A PGC-1*α* inhibitor, SR-18292 (SR), purchased from MedChemExpress (Monmouth Junction, NJ, USA), was dissolved in 10% DMSO and 10% Tween 80 in sterile saline. The vehicle injection consisted of 10% DMSO and 10% Tween 80 in sterile saline.

To determine whether treatment with RTA-408 or ZLN005 has analgesic effects in the CCI model, a single intrathecal (i.t.) injection of different doses of RTA-408 (1, 5, and 10 *μ*g) or ZLN005 (1, 5, and 10 *μ*g) was given on day 7 after CCI. The pain behavior tests were conducted before RTA-408 or ZLN005 injection and at 0 h, 0.5 h, 1 h, 2 h, 4 h, and 8 h after RTA-408 or ZLN005 injection. To determine whether consecutive injections of RTA-408 or ZLN005 can reverse established CCI-induced neuropathic pain, RTA-408 (1, 5, and 10 *μ*g) or ZLN005 (1, 5, and 10 *μ*g) was given once daily for 5 consecutive days (from day 7 to day 11) after SNI establishment. The pain behavior tests were conducted on day 7 and 1 h after RTA-408 or ZLN005 injection each day. To determine whether the Nrf2 inhibitor trigonelline can reverse the analgesic effect of RTA-408, trigonelline (5 *μ*g, i.t.) was given 30 min before RTA-408. The pain behavior tests were performed before trigonelline injection and 0.5 h, 1 h, 2 h, 4 h, and 8 h after RTA-408 injection. To determine whether the PGC-1*α* inhibitor SR-18292 can reverse the analgesic effect of ZLN005, SR-18292 (10 *μ*g, i.t.) was given 30 min before ZLN005. The pain behavior tests were performed before SR-18292 injection and 0.5 h, 1 h, 2 h, 4 h, and 8 h after ZLN005 injection.

To assess the preventive effect of RTA-408 or ZLN005, RTA-408 (10 *μ*g, i.t.) or ZLN005 (10 *μ*g, i.t.) was given once daily from day 0 to day 2 after CCI. The pain behavior tests were performed before CCI and on days 1, 3, 7, and 14.

To determine whether the PGC-1*α* inhibitor SR-18292 can reverse the analgesic effect of RTA-408, SR-18292 (10 *μ*g, i.t.) was given 30 min before RTA-408. The pain behavior tests were performed before trigonelline injection and 0.5 h, 1 h, 2 h, 4 h, and 8 h after RTA-408 injection. The different doses of drugs were chosen based on our preliminary experiments.

### 2.3. Intrathecal Injection

The lumber puncture methods were performed as previously described [23]. In brief, after determining the level of the hip bones with the nondominant hand, the 30-gauge sterile needle was inserted vertically into the L5-6 vertebrae of conscious mice. The needle angle is inclined to approximately 30° when it connects the vertebrae; then, insert the needle into the intervertebral space. An evident reflexive swing of the tail manifested a piercing of the dura, and 5 *μ*L of drugs or vehicles was slowly injected (1 *μ*L/min).

### 2.4. CCI and Behavioral Tests

Neuropathic pain was induced by CCI of the sciatic nerve in this study. Mice were anesthetized with 2% isoflurane via a facemask and were monitored during the surgery. The left common sciatic nerve of each mouse was exposed at the midthigh level, proximal to the trifurcation of the sciatic nerve which was gently freed without stretching muscles and nerves. Three ligatures were loosely tied (5-0 chromic gut sutures) around it and 1 mm apart. In the sham-operated group, the same procedure was performed without injury of the nerves. Mice were randomly divided into the following groups: (1) sham+vehicle group: sham-operated mice with vehicle injection (5 *μ*L, i.t.); (2) CCI+vehicle group: CCI-injured mice with vehicle injection (5 *μ*L, i.t.); (3) CCI+RTA group: CCI-injured mice with RTA-408 injection (1, 5, and 10 *μ*g, i.t.); (4) CCI+Trig group: CCI-injured mice with trigonelline hydrochloride (5 *μ*g, i.t.) injection; (5) CCI+RTA+Trig group: CCI-injured mice with RTA-408 (10 *μ*g, i.t.) combined with trigonelline hydrochloride (5 *μ*g, i.t.) injection; (6) CCI+ZLN group: CCI-injured mice with ZLN005 injection (1, 5, and 10 *μ*g, i.t.); (7) CCI+SR group: CCI-injured mice with SR-18292 injection (10 *μ*g, i.t.); (8) CCI+ZLN+SR group: CCI-injured mice with ZLN005 (10 *μ*g, i.t.) combined with SR-18292 (10 *μ*g, i.t.) injection; and (9) CCI+RTA+SR group: CCI-injured mice with RTA-408 (10 *μ*g, i.t.) combined with SR-18292 (10 *μ*g, i.t.) injection.

Mechanical allodynia was determined by measurement of the paw withdrawal threshold (PWT) using von Frey filaments as previously described [37, 38]. In brief, mice were set in an elevated plexiglass cage for 30 min before the test to adapt to the environment. The tip of filaments was stuck upright to the plantar surface of the left hind paw of each mouse, with each force lasting 5 s. Ascending order of forces (0.02 g, 0.04 g, 0.16 g, 0.4 g, 0.6 g, 1.0 g, 1.4 g, and 2 g) was used, starting with 0.02 g and ending with 2 g. Positive responses were defined as a quick paw lifting or licking. When a positive response was observed, mice were allowed to rest for 5 min before detecting the next descending von Frey filament. PWTs were decided as the lowest force after positive responses.

Thermal hyperalgesia was assessed by measurement of thermal withdrawal latency (TWL) using the Hargreaves plantar test (Ugo Basile, Comerio, VA, Italy) [39]. In brief, mice were placed in transparent separate compartments (10 × 10 × 15 cm) on a glass plate and kept quiet for 30 min before the test. Place the radiant heat source (50% intensity) [40] beneath the plantar surface of the left hind paw. Once the hind paw of mice was moved, the stimulation was shut off and the data was recorded. Experiment with each hind paw was repeated 3 times with a period of 5-6 min intervals. The final TWL was determined from the mean of three measurements. The cutoff time was 20 seconds to avoid tissue damage. The glass plate was cleaned between each interval. Animals exhibiting motor dysfunction were excluded from all the experiments.

### 2.5. Western Blotting

Under deep 2.5% isoflurane anesthesia, the bilateral side of L4-6 spinal cord segments was excised rapidly at different time points. Total tissues were homogenized in cooled RIPA lysis buffer containing a cocktail of protease inhibitors and protein inhibitors on ice. After centrifugation at 12,000 rpm for 30 min at 4°C, the supernatants were collected. The concentration of nuclear and cytoplasmic protein extractions (Nuclear and Cytoplasmic Protein Extraction Kit, Beyotime, Shanghai, China) was measured by using the Bicinchoninic Acid (BCA) Protein Assay Kit (Boster, Wuhan, China) according to the manufacturer's instructions. Then, protein extractions were separated by 10% SDS-PAGE and transferred onto 0.45 *μ*m polyvinylidene fluoride (PVDF) membranes (Millipore, MA, USA). After blocking with 5% nonfat milk or BSA for 1.5 h at RT (room temperature), the membranes were incubated with the following primary antibodies overnight at 4°C: anti-PGC-1*α* (1 : 1000; A11971; ABclonal, Wuhan, China), anti-Nrf2 (1 : 1000; 16369-1-AP; Proteintech, Wuhan, China), anti-NRF-1 (1 : 2000; ab175932; Abcam, Cambridge, UK), anti-TFAM (1 : 500; ab252432; Abcam, Cambridge, UK), and anti-histone H3 (1 : 1000; GB11102; Servicebio, Wuhan, China). After washing with TBST 3 times, the membranes were incubated with a goat anti-rabbit HRP-conjugated secondary antibody (1 : 5000; A21020, Abbkine, Wuhan, China) at RT for 1.5 to 2 h. Finally, the protein expression was detected using the SuperLumia ECL Kit (YEASEN, Shanghai, China) and quantified using chemiluminescence (Bio-Rad, CA, USA). For western blot analysis, the bilateral side of L4-6 spinal cord segments (all laminae) was collected and quantified. The Nrf2 (nuclear) protein was normalized to histone H3, which served as a loading control. The intensity of other proteins (PGC-1*α*, NRF1, and TFAM) was normalized to loading control GAPDH and expressed as the fold of control. The blot density of control groups was set as 1 for quantification.

### 2.6. Immunofluorescence Staining

Under deep 2.5% isoflurane anesthesia, mice were transcranially perfused with 40 mL cold PBS followed by 40 mL 4% cold paraformaldehyde (PFA). The L4-6 segments of the spinal cord were dissected out and postfixed in 4% PFA overnight at 4°C. Spinal cord sections were crosscut into 20 *μ*m on a cryostat (CM1900, Leica, Heidelberg, Germany) after being frozen overnight at -80°C. After being penetrated with 0.3% TritonX-100 for 40 min, sections were blocked with 10% donkey serum for 40 min at RT. Then, they were incubated in the following primary antibodies overnight at 4°C: anti-Nrf2 (1 : 50; AF70006; Affinity, Wuhan, China), anti-neuronal nuclei antibody (1 : 100; NeuN; ab104224, Abcam, Cambridge, UK), anti-glial fibrillary acidic protein antibody (1 : 300; GFAP; 3670; Cell Signaling Technology, MA, USA), and anti-Iba1 antibody (1 : 100; ab5076; Abcam, Cambridge, UK). After washing 5 times with PBST, the sections were incubated with a mixture of Alexa 488-conjugated donkey anti-rabbit secondary antibody (1 : 100; 711-547-003; Jackson ImmunoResearch, PA, USA), Alexa 594-conjugated donkey anti-mouse secondary antibody (1 : 200; 715-585-150; Jackson ImmunoResearch, PA, USA), or Alexa 594-conjugated donkey anti-goat secondary antibody (1 : 200; 705-585-003; Jackson ImmunoResearch, PA, USA) for 2 h at RT. After being washed 5 times with PBST, the sections were then detected with a fluorescence microscope (DP70, Olympus, Japan). As described previously [41, 42], the immunofluorescence intensity was quantified from the ipsilateral spinal cord dorsal horn and calculated by using ImageJ (National Institutes of Health, MD, USA). Immunoreactivity was quantified by calculating immunostaining percentage ([positive immunofluorescent surface area]/[total measured picture area] × 100). Five sections of the spinal cord were randomly selected from each mouse, and one spinal section was analyzed per spinal cord segmental level (L4-L6). Three mice of each group were used for statistical analysis.

### 2.7. Quantification of Mitochondrial DNA (mtDNA) Copy Number

Under deep 2.5% isoflurane anesthesia, the L4-6 spinal cord of mice was collected. The DNA was extracted using the Tissue Genomic DNA Extraction Kit (EP007, ELK Biotechnology, Wuhan, China) according to the manufacturer's instructions. qPCR was performed with SYBR Green PCR SuperMix (ELK Biotechnology, EQ001, Wuhan, China). Quantitative PCR was performed using the 2^−ΔΔCt^ method for the mitochondrial gene ND1 (mtND1) and nuclear-encoded gene *β*-actin. The ratio of mtND1 to *β*-actin represented the relative mtDNA copy number. The following primer sequences were used for PCR:

mtND1: sense: 5′-AACGTAGAATACGCAGCCGG-3′, antisense: 5′-TTGATCGTAACGGAAGCGTG-3′

*β*-Actin sense: 5′-CTTCTATGAGCTGAGTCTCCCTTG-3′, antisense: 5′-GACAGGGGCTCCACTTAGACC-3′

### 2.8. Measurement of ATP Concentrations

Under deep 2.5% isoflurane anesthesia, whole spinal cord sections (L4-L6) were collected. Total ATP production was measured using the ATP assay kit (Nanjing Jiancheng Bioengineering Institute, Nanjing, China). Tissue was washed in cold PBS and resuspended in ATP assay buffer. After centrifugation at 4000 rpm for 5 min, the supernatants were collected and incubated with the ATP probe. Absorbance was monitored at 636 nm with an ultraviolet spectrophotometer (Philes, Nanjing, China).

### 2.9. Statistical Analysis

Data are expressed as means ± SEM and analyzed with GraphPad Prism version 6.0. Western blot results were analyzed using one-way ANOVA followed by the Bonferroni post hoc test. Behavioral data (including PWT and TWL) was analyzed by two-way ANOVA with repeated measures, followed by Bonferroni post hoc analysis. *P* values less than 0.05 were considered statistically significant. The investigator responsible for data analysis was blinded to the experimental design.

## 3. Results

### 3.1. RTA-408 Attenuated CCI-Induced Pain Behaviors

**Figure 1:**
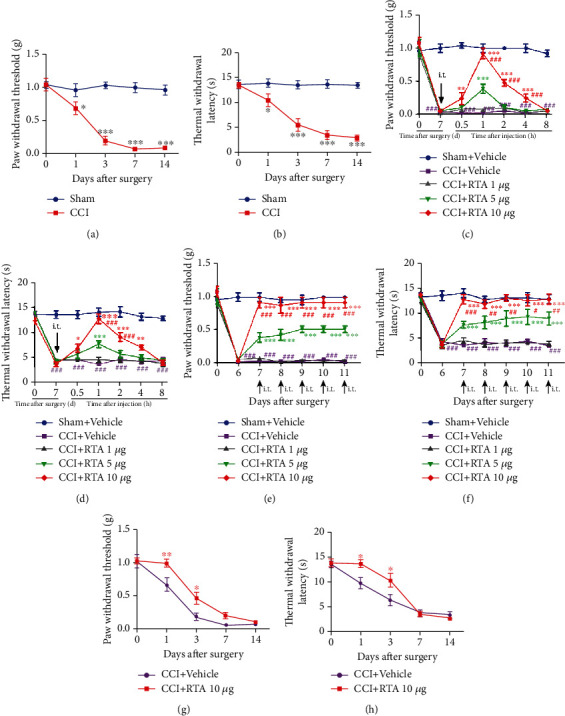
Effect of RTA-408 on the treatment and prevention of mechanical pain and hyperalgesia after chronic constriction injury (CCI). (a, b) Mechanical allodynia and thermal hyperalgesia were used to detect the paw withdrawal threshold (PWT) and thermal withdrawal latency (TWL). PWL and PWT were conducted before CCI (day 0) and at day 1, day 3, day 7, and day 14 after surgery (^∗^*P* < 0.05, ^∗∗^*P* < 0.01, and ^∗∗∗^*P* < 0.001 compared with the sham group, *n* = 5 per group). (c, d) A single injection of RTA-408 (RTA, 1 *μ*g, 5 *μ*g, and 10 *μ*g/5 *μ*L, i.t.) or vehicle (5 *μ*L) was given on day 7 following CCI (^∗^*P* < 0.05, ^∗∗^*P* < 0.01, and ^∗∗∗^*P* < 0.001 compared with the CCI+vehicle group, ^###^*P* < 0.001 compared with the group treated with RTA-408 (5 *μ*g)). In contrast, the CCI+vehicle group had no significant change in PWT and TWL (^###^*P* < 0.001 compared with the sham+vehicle group). No significant difference in the baseline thresholds was observed among all groups (*n* = 5 per group). (e, f) RTA-408 (1 *μ*g, 5 *μ*g, and 10 *μ*g/5 *μ*L, i.t.) or vehicle (5 *μ*L) was given for 5 consecutive days from day 7 to day 11 (^∗∗∗^*P* < 0.001 compared with the CCI+vehicle group, ^#^*P* < 0.05, ^##^*P* < 0.01, and ^###^*P* < 0.001 compared with the group treated with RTA-408 (5 *μ*g)). In contrast, the CCI+vehicle group had no significant change in PWT and TWL (^###^*P* < 0.001 compared with the sham+vehicle group) (*n* = 5 per group). (g, h) Preventive effect of RTA-408 on the development of pain hypersensitivity following CCI. RTA-408 (10 *μ*g, i.t.) was given once daily from day 0 to day 2 after CCI. The pain behavioral tests were performed before CCI and on day 1, day 3, day 7, and day 14 after CCI. Two-way ANOVA with repeated measures was performed, followed by the Bonferroni post hoc test (^∗^*P* < 0.05 compared with CCI+vehicle mice, *n* = 5 per group).

Before surgery, the von Frey test showed no significant differences in PWT among all groups. After CCI, PWT in the ipsilateral paws potently decreased from day 3 to day 14. The most severe decrease in PWT appeared on day 7 after CCI (Figure 1(a)). Likely, the Hargreaves plantar test showed no significant difference in TWL among all groups before surgery. The ipsilateral TWL potently attenuated from day 3 after surgery and remained on day 14 (Figure 1(b)). There was no significant change in PWT and TWL in the sham group during the experiment. These results indicated that CCI induced a marked decrease in PWT and TWL in a pattern of a rapid-onset and long-lasting manner.

To assess the analgesic effect of RTA-408, a single injection of different doses of RTA-408 (1 *μ*g, 5 *μ*g, and 10 *μ*g, i.t.) or vehicle (5 *μ*L, i.t.) was given on day 7 after CCI. PWT and TWL tests were performed at 0 h, 0.5 h, 1 h, 2 h, 4 h, and 8 h after RTA-408 injection. As shown in Figures 1(c) and 1(d), administration of 1 *μ*g RTA-408 had no significant influence on PWT and TWL. However, 5 *μ*g or 10 *μ*g RTA-408 significantly reversed the mechanical allodynia and thermal hyperalgesia beginning at 0.5 h, peaking at 1 h, and lasting at least 4 h. Besides, there were significant differences between the 5 *μ*g and 10 *μ*g RTA-408, suggesting that i.t. RTA-408 could reverse mechanical allodynia and thermal hyperalgesia in a dose-dependent manner after CCI. In contrast, CCI or sham mice treated with vehicle alone showed no significant change in PWT and TWL. Moreover, after CCI, repeated i.t. administration of RTA-408 to mice from day 7 to day 11 significantly reversed the established mechanical allodynia and thermal hyperalgesia without showing any signs of tolerance (Figures 1(e) and 1(f)).

### 3.2. Preventive Effect of RTA-408 on the Development of CCI-Induced Pain Hypersensitivity

To determine the preventive effect of RTA-408, 10 *μ*g RTA-408 was i.t. administered once daily from day 0 to day 2. The pain behaviors were tested before CCI and on day 1, day 3, day 7, and day 14 following CCI. As shown in Figures 1(g) and 1(h), the PWT and TWL were significantly upregulated on day 1 and day 3, but not on day 7 and day 14. These results suggest that early administration of RTA-408 (10 *μ*g, i.t.) from day 0 to day 2 delayed the onset of CCI-induced mechanical allodynia and thermal hyperalgesia.

### 3.3. Expression and Cellular Localization of Spinal Nrf2 after CCI

**Figure 2:**
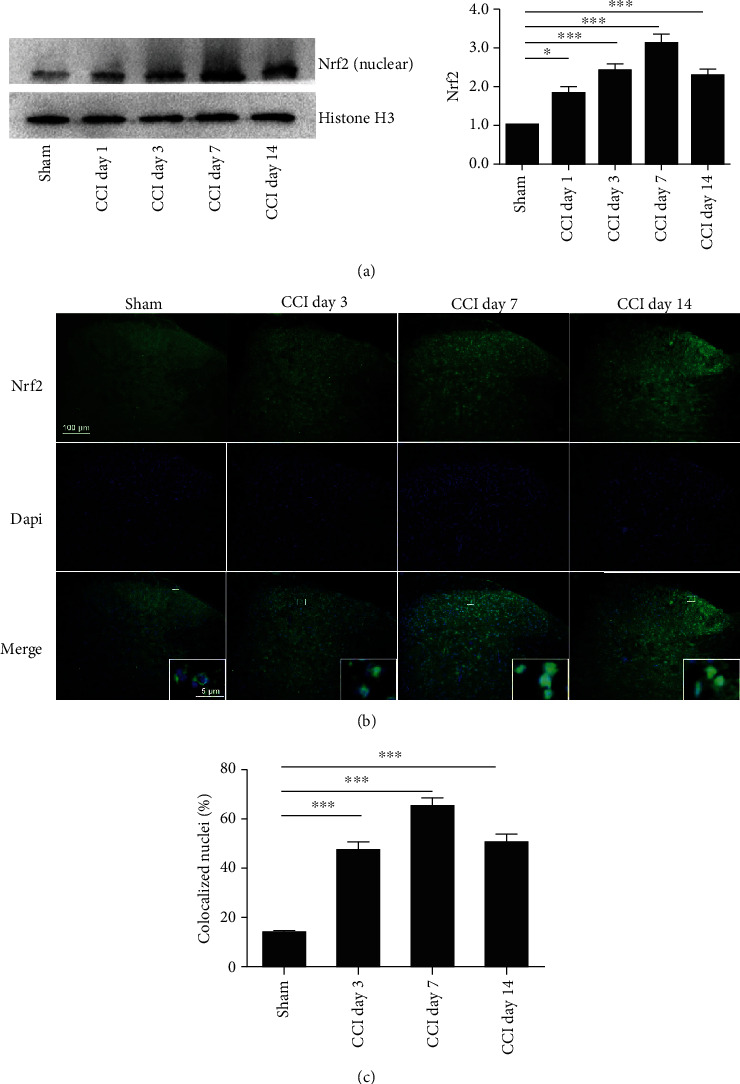
Time course of Nrf2 expression in the spinal cord after CCI. (a) Western blotting showed the time course of Nrf2 protein in the nuclear extracts. Representative blots and blot density (normalized to histone H3 loading control) are presented (^∗^*P* < 0.05, ^∗∗∗^*P* < 0.001 compared with the sham group, *n* = 5 per group). (b) Representative fluorescent photomicrographs showed the time course expression of Nrf2 and nuclei in the ipsilateral spinal cord dorsal horn following CCI. One-way ANOVA followed by the Bonferroni post hoc test was used for western blot results and immunochemistry data. Magnification: 100x and 2000x (insets) (scale bar = 100 *μ*m). (c) The number of DAPI-positive nuclei colocalized with Nrf2 is expressed as a ratio of the total number of nuclei in the spinal cord dorsal horn. One-way ANOVA followed by Bonferroni analysis was used to test the differences among groups (^∗∗∗^*P* < 0.001 compared with the sham group, *n* = 3 per group).

Western blot analysis and immunohistochemistry were performed to determine the activation of Nrf2 in the spinal cord following CCI. As shown in Figure 2(a), the expression of Nrf2 protein in the nuclear extracts was rapidly increased, beginning at day 1, and remained at high levels until the last observation on day 14 in the spinal cord dorsal horn following CCI. We then detect the colocalization of Nrf2 with the nucleus using immunofluorescence staining (Figure 2(b)). As predicted, the proportion of Nrf2 nuclei was increased following CCI in a time-dependent fashion (Figure 2(c)), which is consistent with our western blot data.

### 3.4. Effects of RTA-408 on Nrf2 Signaling in the Spinal Cord of CCI Mice

**Figure 3:**
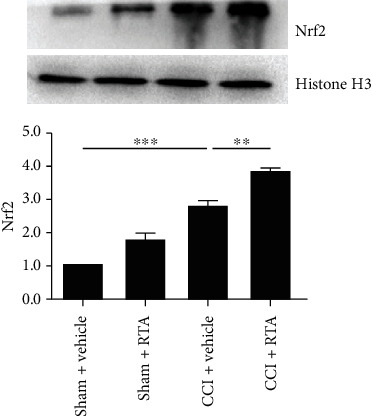
Effect of RTA-408 on the spinal expression of Nrf2 in the nuclear extracts of CCI mice. The spinal expression of Nrf2 in the nuclear extracts was significantly increased following CCI. RTA-408 administration further increased the spinal expression of Nrf2 in the nuclear extracts in the sham group and CCI group. Representative blots and blot density (normalized to histone H3 loading control) are presented. One-way ANOVA followed by the Bonferroni post hoc test was used for western blot results (^∗∗^*P* < 0.01, ^∗∗∗^*P* < 0.001 compared with the indicated group, *n* = 5 per group).

To determine whether RTA-408 could activate Nrf2 following CCI in the spinal cord, we treated CCI mice and sham mice with RTA-408 (10 *μ*g, i.t.) once a day from day 7 to day 11 and examined the expression of Nrf2 protein in the nuclear extracts after the final administration. As shown in Figure 3, the expression of Nrf2 protein in the nuclear extracts was rapidly enhanced after RTA-408 administration in the CCI group. These results demonstrated that RTA-408 could significantly activate Nrf2 following CCI in the spinal cord.

**Figure 4:**
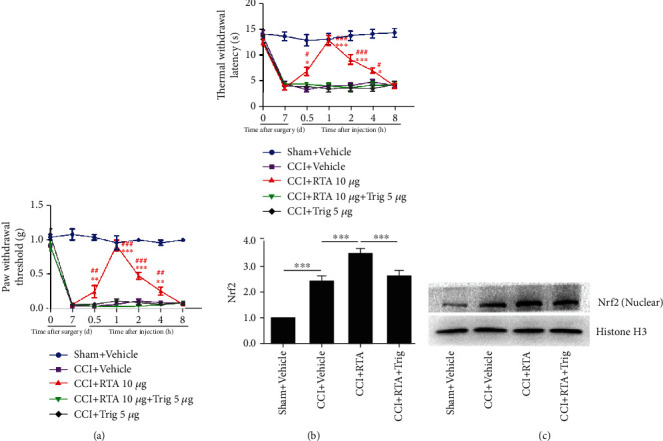
Effect of the Nrf2 inhibitor on pain hypersensitivity and the spinal expression of Nrf2 in the nuclear extracts. (a, b) The analgesic effect of RTA-408 (10 *μ*g, i.t.) in CCI mice was completely inhibited by the Nrf2 inhibitor trigonelline (Trig). Two-way ANOVA with repeated measures was performed, followed by the Bonferroni post hoc test (^∗^*P* < 0.05, ^∗∗^*P* < 0.01, and ^∗∗∗^*P* < 0.001 compared with the sham+vehicle group, ^#^*P* < 0.05, ^##^*P* < 0.01, and ^###^*P* < 0.001 compared with the CCI+RTA+Trig group, *n* = 5 per group). (c) The spinal expression of Nrf2 in the nuclear extracts was significantly increased following CCI. Representative blots and blot density (normalized to histone H3 loading control) are presented (^∗∗∗^*P* < 0.001 compared with the sham+vehicle group). RTA-408 administration further increased the spinal expression of Nrf2 in the nuclear extracts in the CCI group (^∗∗∗^*P* < 0.001 compared with the CCI+vehicle group, *n* = 5 in each group), which was significantly inhibited by the preinjection of trigonelline. One-way ANOVA followed by Bonferroni analysis was used to test the differences among groups (^∗∗∗^*P* < 0.001 compared with the CCI+RTA-408 group, *n* = 5 per group).

To evaluate whether activation of the Nrf2 signaling pathway is responsible for the antinociceptive effects of RTA-408, the Nrf2 inhibitor trigonelline was given 30 min before RTA-408 administration on day 7 following CCI. The threshold of PWT and TWL was tested before trigonelline administration and at 0.5 h, 1 h, 2 h, 4 h, and 8 h after administration [43]. As shown in Figures 4(a) and 4(b), the alleviating effect of RTA-408 against CCI-induced pain hypersensitivity was completely prevented by trigonelline injection. Trigonelline did not change mechanical allodynia and thermal hyperalgesia in mice without RTA-408 treatment in the CCI group.

**Figure 5:**
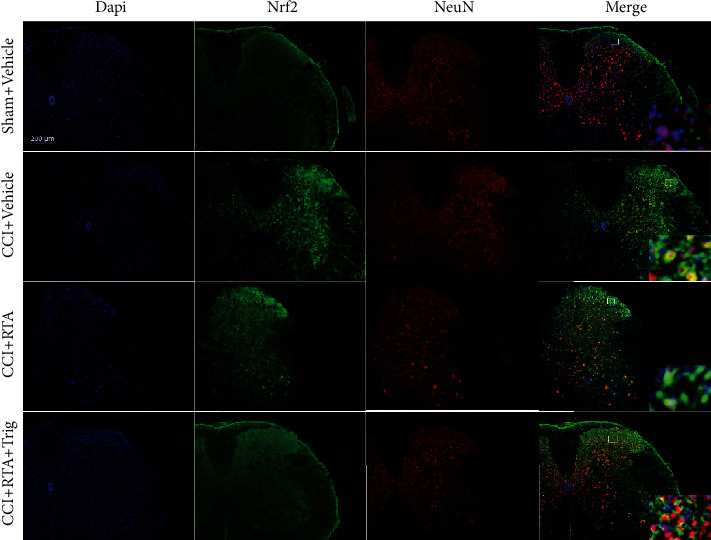
Distribution of Nrf2 in the spinal cord following CCI. Nrf2 was mainly colocalized with neurons (NeuN, a neuronal marker, red) in the ipsilateral spinal cord dorsal horn. Magnification: 100x and 1000x (insets) (scale bar = 200 *μ*m, *n* = 3 per group).

We next examined the activity of Nrf2 following trigonelline coinjection. As shown in Figure 4(c), the activity of Nrf2 enhanced by RTA-408 was significantly suppressed by trigonelline treatment. Consistent with our western blot data, the immunofluorescence data showed that RTA-408 injection could further enhance Nrf2 activation following CCI, and cotreatment with trigonelline reduced the activation of Nrf2 in the spinal cord dorsal horn compared with RTA-408 treatment alone following CCI (Figure 5). Moreover, our double immunofluorescence staining showed that Nrf2 was mainly colocalized with neurons and a minority in microglia in the spinal cord dorsal horn (Figure 5, Supplementary Figure 1, and Supplementary Figure 2). These results suggested that RTA-408 could alleviate CCI-induced pain hypersensitivity through activation of Nrf2 in the spinal cord.

### 3.5. RTA-408 Inhibited Microglia and Astrocyte Activation in the Spinal Cord Dorsal Horn of CCI Mice

GFAP level analysis showed that the reactive astrocytes were robustly activated in the ipsilateral spinal cord dorsal horn following CCI surgery. Moreover, the astrocytes exhibited robust proliferation and hypertrophy with increased podocytic processes (Supplementary Figure 1). Iba1 level analysis showed that the reactive microglia were significantly activated in the ipsilateral spinal cord dorsal horn following CCI surgery. Additionally, the microglia were robustly activated and clustered, with bulk somata and relatively short processes (Supplementary Figure 2).

To investigate the effects of RTA-408 on spinal cord astrocytes and microglia, we treated CCI mice with RTA-408 or vehicle. The results showed that RTA-408 injection almost completely blocked CCI-induced astrocyte activation in the spinal cord dorsal horn. Moreover, RTA-408 treatment also inhibited CCI-induced microglia activation. Furthermore, all these protective effects of RTA-408 could be blocked by the Nrf2 inhibitor (Supplementary Figure 1 and Supplementary Figure 2). These results indicated that Nrf2 activation has the ability to reverse nerve injury-induced astrogliosis and microglia activation in the spinal cord dorsal horn.

### 3.6. Mitochondrial Biogenesis and Mitochondrial Function in the Spinal Cord of CCI Mice

**Figure 6:**
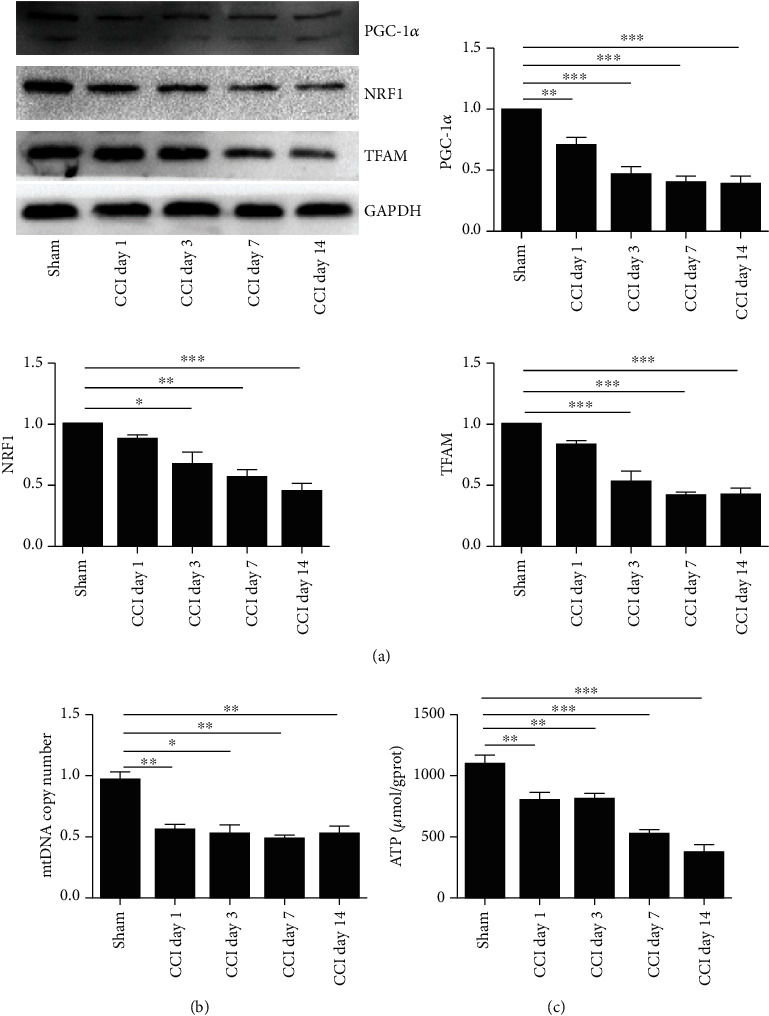
Time course of mitochondrial protein and mtDNA content in the spinal cord following CCI. The spinal cord was collected and analyzed for (a) mitochondrial protein expression, (b) mtDNA content, and (c) ATP levels following CCI. The intensity of protein blots was normalized to loading control GAPDH antibody and expressed as the fold of control. One-way ANOVA followed by Bonferroni analysis was used to test the differences among groups (^∗^*P* < 0.05, ^∗∗^*P* < 0.01, and ^∗∗∗^*P* < 0.001 compared with the sham group).

An imbalance of redox biology disrupts mitochondrial bioenergetics, which is thought to lead to neuropathic pain [44, 45]. PGC-1*α* is the principal regulatory factor of MB and is involved in the transcriptional control of several mitochondrial genes, including NRF1 and TFAM [16]. Thus, the time course (day 1, day 3, day 7, and day 14) of CCI was conducted to test the expression of the PGC-1*α*/NRF1/TFAM gene and mtDNA copy number in the spinal cord. Western blot analysis showed that the expression of PGC-1*α* was significantly diminished at day 1 in the dorsal horn of the spinal cord following CCI. Moreover, the expression of NRF1 and TFAM was decreased from day 3 to day 14 (Figure 6(a)). To investigate the changes of mtDNA expression in the spinal cord of CCI mice, the L4-6 spinal cord was collected on day 1, day 3, day 7, and day 14 after CCI. RT-PCR results showed that expression of mtDNA downregulated from day 1 compared to that of sham mice, which is consistent with changes in mitochondrial genes (Figure 6(b)). Furthermore, the time course (day 1, day 3, day 7, and day 14) of ATP contents was measured (Figure 6(c)). The level of ATP was significantly reduced from day 1 in the dorsal horn of the spinal cord following CCI. These results indicated that mitochondrial dysfunction was induced by CCI in the spinal cord.

### 3.7. PGC-1*α* Activator Attenuated CCI-Induced Pain Behaviors

**Figure 7:**
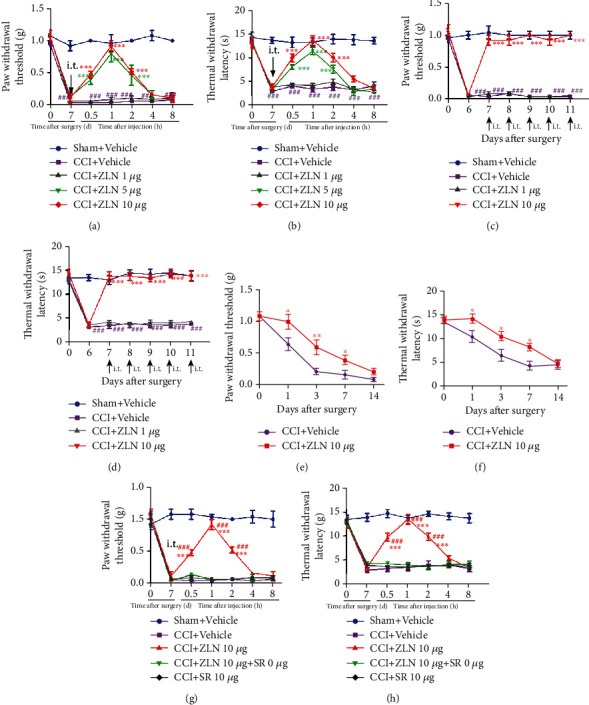
Effect of ZLN005 on the treatment and prevention of mechanical pain and hyperalgesia after CCI. (a, b) A single injection of ZLN005 (1 *μ*g, 5 *μ*g, and 10 *μ*g/5 *μ*L, i.t.) or vehicle (5 *μ*L) was given on day 7 following CCI (^∗∗∗^*P* < 0.001 compared with the CCI+vehicle group). In contrast, the CCI+vehicle group had no significant change in PWT and TWL (^###^*P* < 0.001 compared with the sham+vehicle group). No significant difference in the baseline thresholds was observed among all groups (*n* = 5 per group). (c, d) ZLN005 (1 *μ*g and 10 *μ*g/5 *μ*L, i.t.) or vehicle (5 *μ*L) was given for 5 consecutive days from day 7 to day 11. Treatment with ZLN005 (10 *μ*g) significantly reversed PWT and TWL in CCI mice (^∗∗∗^*P* < 0.001 compared with the CCI+vehicle group). In contrast, the CCI+vehicle group had no significant change in PWT and TWL (^###^*P* < 0.001 compared with the sham+vehicle group) (*n* = 5 per group). (e, f) Preventive effect of ZLN005 on the development of CCI. ZLN005 (10 *μ*g, i.t.) was given once daily from day 0 to day 2 after CCI. The pain behavioral tests were performed before CCI and on day 1, day 3, day 7, and day 14 after CCI. Treatment with ZLN005 (10 *μ*g, i.t.) significantly elevated the PWT and TWL at day 3 and day 7 after CCI. However, no significant difference was observed on day 14 (^∗^*P* < 0.05, ^∗∗^*P* < 0.01, and ^∗∗∗^*P* < 0.001 compared with CCI+vehicle mice) (*n* = 5 per group). (g, h) The analgesic effect of ZLN005 in CCI mice was completely inhibited by the PGC-1*α* inhibitor SR-18292 (SR). Two-way ANOVA with repeated measures was performed, followed by the Bonferroni post hoc test (^∗∗∗^*P* < 0.001 compared with the sham+vehicle group, ^###^*P* < 0.001 compared with the CCI+ZLN+SR group, *n* = 5 per group).

As the main regulatory factor of MB, PGC-1*α* was significantly decreased following CCI. To further assess the role of PGC-1*α* in CCI-induced pain model, a single injection of the PGC-1*α* activator ZLN005 (1 *μ*g, 5 *μ*g, and 10 *μ*g, i.t.) and vehicle (5 *μ*L) was given on day 7 after CCI. PWT and TWL tests were performed at 0 h, 0.5 h, 1 h, 2 h, 4 h, and 8 h after RTA-408 injection. As shown in Figures 7(a) and 7(b), a single i.t. injection of ZLN005 (5 *μ*g and 10 *μ*g) significantly increased mechanical allodynia and thermal hyperalgesia following CCI beginning at 0.5 h, peaking at 1 h, and lasting for at least 2 h. However, there was no significant difference between the two groups (5 *μ*g and 10 *μ*g). To determine the accumulative effect of the PGC-1*α* activator, ZLN005 (1 *μ*g and 10 *μ*g) was i.t. injected from day 7 to day 11. As shown in Figures 7(c) and 7(d), repeated injection of ZLN005 resulted in significant reversal of mechanical and thermal hyperalgesia without signs of tolerance after CCI. These results suggested that ZLN005 significantly reversed mechanical allodynia and thermal hyperalgesia following CCI.

### 3.8. Preventive Effect of the PGC-1*α* Activator on the Development of CCI-Induced Pain Hypersensitivity

To determine the preventive effect of ZLN005, ZLN005 (10 *μ*g, i.t.) was administered once daily from day 0 to day 2. The pain behaviors were tested before CCI and on day 1, day 3, day 7, and day 14 following surgery. As shown in Figures 7(e) and 7(f), mice pretreated with ZLN005 showed upregulated PWT and TWL on day 1, day 3, and day 7, but not day 14. These results suggested that early administration of ZLN005 (10 *μ*g, i.t.) significantly delayed the onset of CCI-induced mechanical allodynia and thermal hyperalgesia.

To evaluate whether PGC-1*α* is involved in the antinociceptive effects of ZLN005, the PGC-1*α* inhibitor SR-18292 was given 30 min before ZLN005 administration on day 7 following CCI. The threshold of PWT and TWL was tested before SR-18292 administration and at 0.5 h, 1 h, 2 h, 4 h, and 8 h after ZLN005 administration. As shown in Figures 7(g) and 7(h), the effect of ZLN005 against CCI-induced pain hypersensitivity was completely prevented by SR-18292 injection. SR-18292 did not change mechanical allodynia and thermal hyperalgesia in mice following CCI.

### 3.9. Effect of RTA-408 on Mitochondrial Biogenesis in the Spinal Cord of CCI Mice

**Figure 8:**
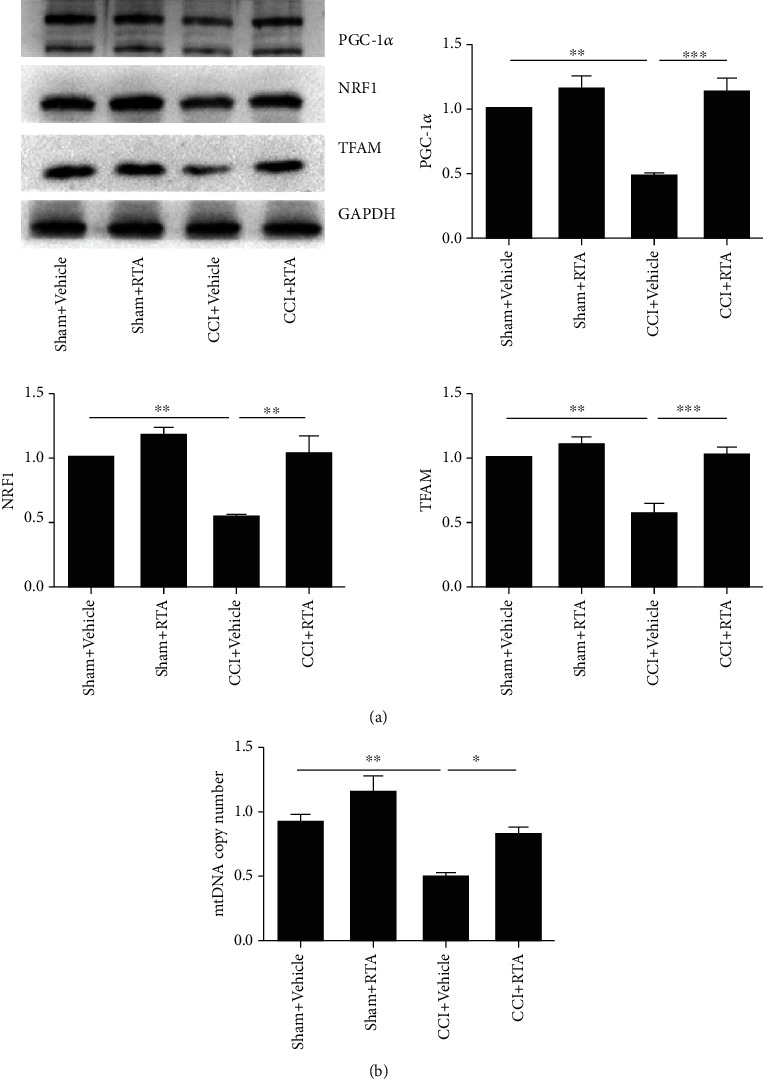
Effect of RTA-408 on mitochondrial protein and mtDNA content and on the spinal cord following CCI. The spinal cord was extracted and analyzed for (a) mitochondrial protein expression of PGC-1*α*, NRF1, and TFAM and (b) mtDNA content. The intensity of protein blots was normalized to a loading control GAPDH antibody and expressed as the fold of control. One-way ANOVA followed by Bonferroni analysis was used to test the differences among groups (^∗^*P* < 0.05, ^∗∗^*P* < 0.01, and ^∗∗∗^*P* < 0.001 compared with the indicated group, *n* = 5 per group).

**Figure 9:**
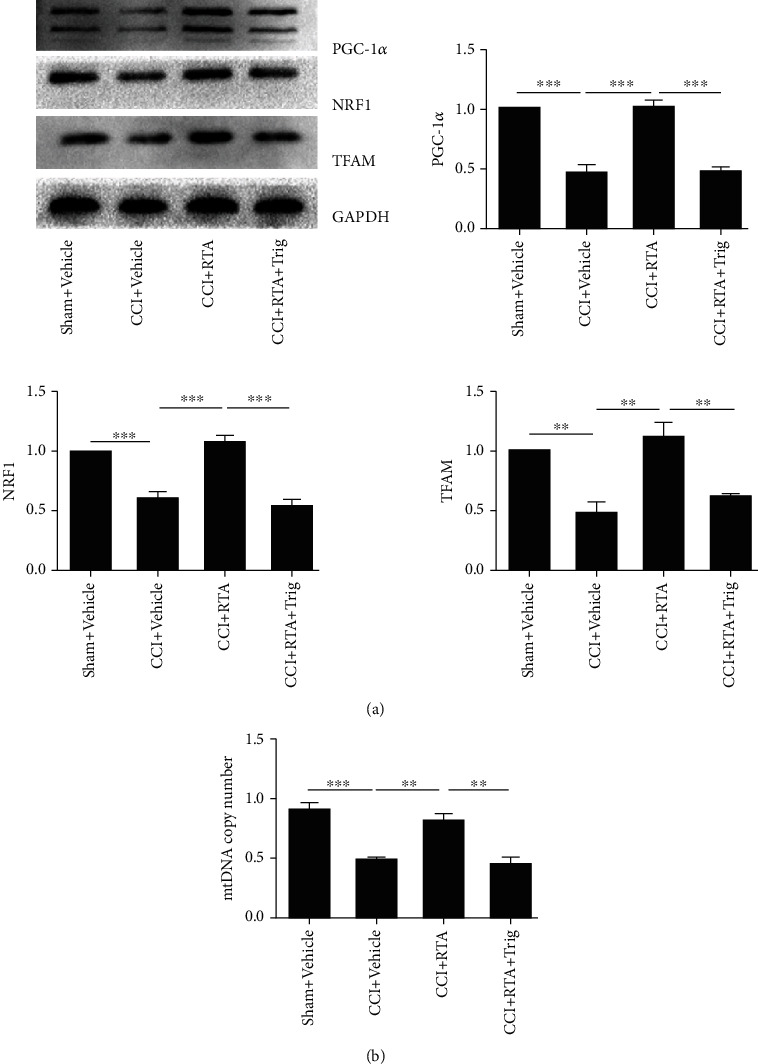
Effect of trigonelline on mitochondrial protein and mtDNA in the spinal cord following CCI. The spinal cord was extracted and analyzed for (a) mitochondrial protein expression of PGC-1*α*, NRF1, and TFAM and (b) mtDNA content. The intensity of protein blots was normalized to a loading control GAPDH antibody and expressed as the fold of control. One-way ANOVA followed by Bonferroni analysis was used to test the differences among groups (^∗∗^*P* < 0.01, ^∗∗∗^*P* < 0.001 compared with the indicated group, *n* = 5 per group).

To determine the effect of RTA-408 on MB in the spinal cord of CCI mice, the L4-6 spinal cord was extracted after the last injection of RTA-408 (10 *μ*g) on day 11 and examined for mitochondrial genes and mtDNA expression. As shown in Figure 8(a), treatment with RTA-408 for 5 consecutive days (from day 7 to day 11) significantly reversed the downregulation of PGC-1*α*/NRF1/TFAM compared to the CCI group. Moreover, RTA-408-treated mice completely restored the downregulation of mtDNA in the spinal cord caused by CCI (Figure 8(b)). However, RTA-408 had no significant effect on mtDNA in the sham group. Further, trigonelline significantly reversed the effect of RTA-408 in decreasing the expression of PGC-1*α*/NRF1/TFAM, as well as downregulating the expression of mtDNA (Figures 9(a) and 9(b)). These results suggested that RTA-408 treatment could restore mitochondrial bioenergetics via the Nrf2 signaling pathway in the CCI model.

### 3.10. PGC-1*α* Was Required for RTA-408 Induction of Mitochondrial Biogenesis

**Figure 10:**
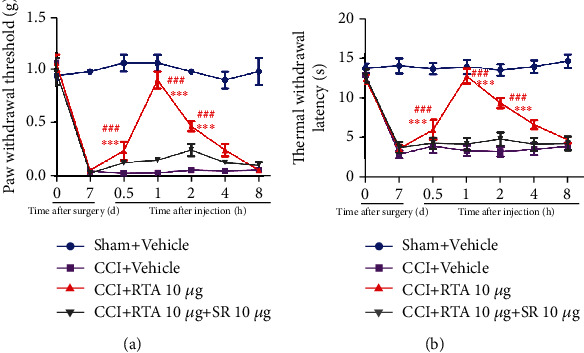
Effect of the PGC-1*α* inhibitor on the analgesic effect of RTA-408. The analgesic effect of RTA-408 in CCI mice was completely inhibited by the PGC-1*α* inhibitor SR-18292. Two-way ANOVA with repeated measures was performed, followed by the Bonferroni post hoc test (^∗∗∗^*P* < 0.001 compared with the sham+vehicle group, ^###^*P* < 0.001 compared with the CCI+RTA+SR group, *n* = 5 per group).

We have shown in previous studies that RTA-408 can restore PGC-1*α* expression following CCI. To determine whether Nrf2 could activate PGC-1*α* in the spinal cord following CCI, the PGC-1*α* inhibitor SR-18292 was given 30 min before RTA-408 administration on day 7 following CCI. The threshold of PWT and TWL was tested before SR-18292 administration and at 0.5 h, 1 h, 2 h, 4 h, and 8 h after RTA-408 administration. As shown in Figures 10(a) and 10(b), the effect of RTA-408 against CCI-induced pain hypersensitivity was completely inhibited by SR-18292 injection. SR-18292 did not change mechanical allodynia and thermal hyperalgesia in the CCI group. These results suggested that Nrf2 could activate PGC-1*α* in the spinal cord following CCI.

## 4. Discussion

This study demonstrated that (1) i.t. injection of a novel synthetic triterpenoid RTA-408 significantly reversed the mechanical allodynia and thermal hyperalgesia in a dose-dependent manner in CCI mice; (2) Nrf2 was significantly activated by CCI and further activated after RTA-408 treatment in the spinal cord; (3) persistent mitochondrial dysfunction was induced by CCI in the spinal cord in a time-dependent manner; (4) activation of PGC-1*α* significantly inhibited the mechanical allodynia and thermal hyperalgesia in CCI mice; and (5) RTA-408 treatment restored MB in the spinal cord of CCI mice, which was reversed by the PGC-1*α* inhibitor. Together, these results suggested that RTA-408 may induce MB through the Nrf2 signaling pathway for the treatment of neuropathic pain in a PGC-1*α*-dependent manner in the spinal cord.

A series of studies have shown that the production of reactive oxygen species (ROS) leads to the induction of neuropathic pain [46, 47]. Our previous study and others have shown that NADPH oxidase 2- (Nox2-) derived ROS production plays a critical role in cancer-induced bone pain and neuropathic pain [11, 48]. Nrf2 is a key regulator which suppresses oxidative stress. Once activated, the Keap1-Nrf2 complex was dissociated to facilitate Nrf2 translocation to the nucleus, thus regulating the transcription of antioxidant-related genes [17–19]. Our previous study has reported the crucial role of Nrf2 in treating paclitaxel-induced neuropathic pain [23]. In this study, we first examined the analgesic effects of RTA-408, which could be reversed by the Nrf2 inhibitor. These data demonstrated that activation of Nrf2 contributes to the analgesic effects of RTA-408.

Moreover, we found that the proportion of Nrf2^+^ nuclei in CCI groups was significantly elevated in the spinal cord and Nrf2 protein levels in the nuclear extracts were robustly increased. These results indicated the CCI-induced rapid activation of the endogenous antioxidant defense system. Additionally, we found that RTA-408 could further promote Nrf2 translocation to the nucleus and significantly inhibited the activation of astrocytes and microglia. These findings are consistent with our previous study, which demonstrated that another Nrf2 agonist, oltipraz, plays an essential role in inhibiting the activation of astrocytes and microglia [23]. However, Li et al. [43] illustrated that Nrf2 was only modestly activated in the DRGs of SNI models. This discrepancy may be due to the use of different tissues, different pain models, and different animals. In spite of this, they found that activation of Nrf2 could alleviate neuropathic pain, which may be related to the restoration of MB.

Mitochondria are essential organelles for cellular homeostasis, including energy production through oxidative phosphorylation, which are required for ATP synthesis [49, 50]. Moreover, mitochondria are the main places of ROS production and attack. Thus, maintaining a balance between degradation and mitochondrial biogenesis (MB) is necessary to maintain high-quality mitochondria and reduce oxidative stress. Recently, mitochondrial dysfunction has been implicated in numerous neuropathic pain models [9, 51]. However, the exact signaling mechanism mediating MB in neuropathic pain remains unknown. In the present study, we observed that CCI caused a significant decrease in several mitochondrial genes, including PGC-1*α*, NRF1, and TFAM, and mtDNA copy number in a time-dependent manner. Consistent with reduced mitochondrial contents, the ATP levels were significantly decreased in the CCI group. The damage of mitochondria directly affects the ATP contents [52], and ATP deficiency can lead to depletion of Na+/K+ ATPase. This may generate hyperexcitability and ectopic activity characteristic of neuropathic pain [53]. Together, these results suggested that CCI-induced impaired mitochondrial biogenesis and function may be the crucial mechanism of neuropathic pain.

Nrf2 is not only a major regulator of redox homeostasis [54] but also involved in the transcriptional control of several mitochondrial genes, including NRF1 and TFAM [16]. It has been reported that Nrf2 participated in MB by activating the ARE/PGC-1*α* signaling pathway [25, 55, 56]. PGC-1*α* is a critical transcriptional coactivator that regulates MB and mitochondrial function [16]. Kashiwagi et al. [57] first showed that recombinant PGC-1*α* (rPGC-1*α*) could increase mechanical allodynia and thermal hyperalgesia in morphine tolerance rats. In this study, we explored the effect of PGC-1*α* in CCI-induced pain mice. Our results showed that repeated injection of the PGC-1*α* activator ZLN005 (10 *μ*g, i.t.) significantly reversed the established pain hypersensitivity in CCI mice. In addition, early administration of ZLN005 (10 *μ*g, i.t.) significantly delayed the onset of CCI-induced mechanical allodynia and thermal hyperalgesia. Moreover, inhibition of spinal PGC-1*α* significantly reversed the analgesic effect of ZLN005, illustrating that PGC-1*α* is the primary target of ZLN005 for the treatment of pain hypersensitivity.

To further explore the role of PGC-1*α*-mediated MB in the development of CCI-induced pain hypersensitivity, mitochondrial proliferative markers (including PGC-1*α*, NRF1, and TFAM) were examined after treatment with RTA-408. As expected, RTA-408 significantly reversed these decreases, notably that in PGC-1*α*. These results indicated that RTA-408 could stimulate MB and improved mitochondria via Nrf2 signaling in the spinal cord of CCI mice. Notably, treatment with RTA-408 has no significant effect on mitochondrial biogenesis in naïve mice but stimulated MB in CCI mice in the spinal cord. Several studies have demonstrated the existence of regulatory loops between Nrf2 and PGC-1*α* in MB [26]. Whether Nrf2 is directly involved in regulating this process is still controversial. Thus, in this study, we preinjected the PGC-1*α* inhibitor 30 min before RTA-408 treatment in the CCI model to assess the mechanical allodynia and thermal hyperalgesia. Data showed that inhibition of spinal PGC-1*α* significantly reversed the analgesic effect of RTA-408, illustrating that RTA-408 stimulated MB in CCI mice through the Nrf2 signaling pathway, and the mechanism was dependent on PGC-1*α*.

In summary, our study provided evidence that RTA-408 could attenuate CCI-induced neuropathic pain via induction of PGC-1*α*-mediated MB in the spinal cord dorsal horn. Although the precise mechanism by which RTA-408 mediates neuropathic pain needs to be further investigated, our findings suggested a possible therapeutic pathway to stimulate MB while improving neuropathic pain hypersensitivity.

## Data Availability

The data that support the findings of the current study are available from the corresponding author upon reasonable request.

## Additional Points

The following chemical compounds were studied in this article: RTA-408 (PubChem CID: 71811910), trigonelline hydrochloride (PubChem CID: 134606), ZLN005 (PubChem CID: 899323), and SR-18292 (PubChem CID: 129896798).

## Supplementary Materials

Supplementary 1 Supplementary Figure 1. (A) Representative images of double immunostaining showing colocalization of GFAP (red) and Nrf2 (green) in the ipsilateral spinal cord dorsal horn (magnification: 100x and 1000x; scale bar = 200 *μ*m and 10 *μ*m). (B) The quantitative data of double immunofluorescence staining. One-way ANOVA followed by Bonferroni analysis was used to test the differences among groups (^∗∗^*P* < 0.01, ^∗∗∗^*P* < 0.001 compared with the indicated group, *n* = 3 per group).

Supplementary 2 Supplementary Figure 2. (A) Representative images of double immunostaining showing colocalization of Iba1 (red) and Nrf2 (green) in the ipsilateral spinal cord dorsal horn (magnification: 100x and 1000x; scale bar = 200 *μ*m and 10 *μ*m). (B) The quantitative data of double immunofluorescence staining. (C) Double immunofluorescence staining showed that Nrf2 was mainly expressed in neurons but not in astrocyte cells in the spinal cord dorsal horn in CCI mice. Yellow represents colocalization. One-way ANOVA followed by Bonferroni analysis was used to test the differences among groups (^∗∗^*P* < 0.01, ^∗∗∗^*P* < 0.001 compared with the indicated group, *n* = 3 per group).
