# Supplementary figures and images for: Nrf2 Activation Attenuates Chronic Constriction Injury-Induced Neuropathic Pain via Induction of PGC-1α-Mediated Mitochondrial Biogenesis in the Spinal Cord
Source: Oxid Med Cell Longev. 2021 Oct 21;2021:9577874. doi: 10.1155/2021/9577874 (PMC8554522; doi:10.1155/2021/9577874)

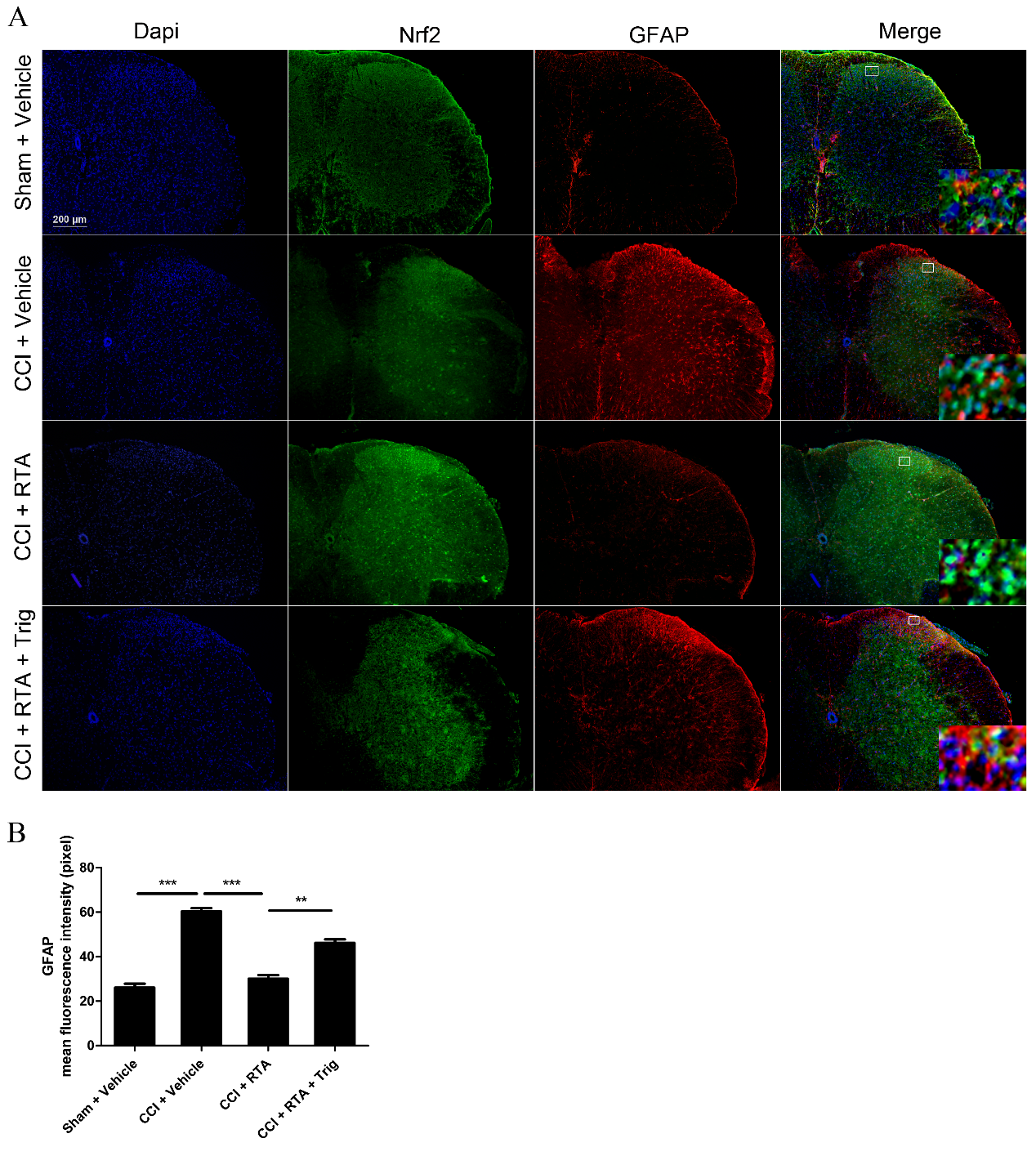

Supplement: Supplementary 1 — Supplementary Figure 1. (A) Representative images of double immunostaining showing colocalization of GFAP (red) and Nrf2 (green) in the ipsilateral spinal cord dorsal horn (magnification: 100x and 1000x; scale bar = 200 μm and 10 μm). (B) The quantitative data of double immunofluorescence staining. One-way ANOVA followed by Bonferroni analysis was used to test the differences among groups (∗∗P < 0.01, ∗∗∗P < 0.001 compared with the indicated group, n = 3 per group). [file 9577874.f1.docx]

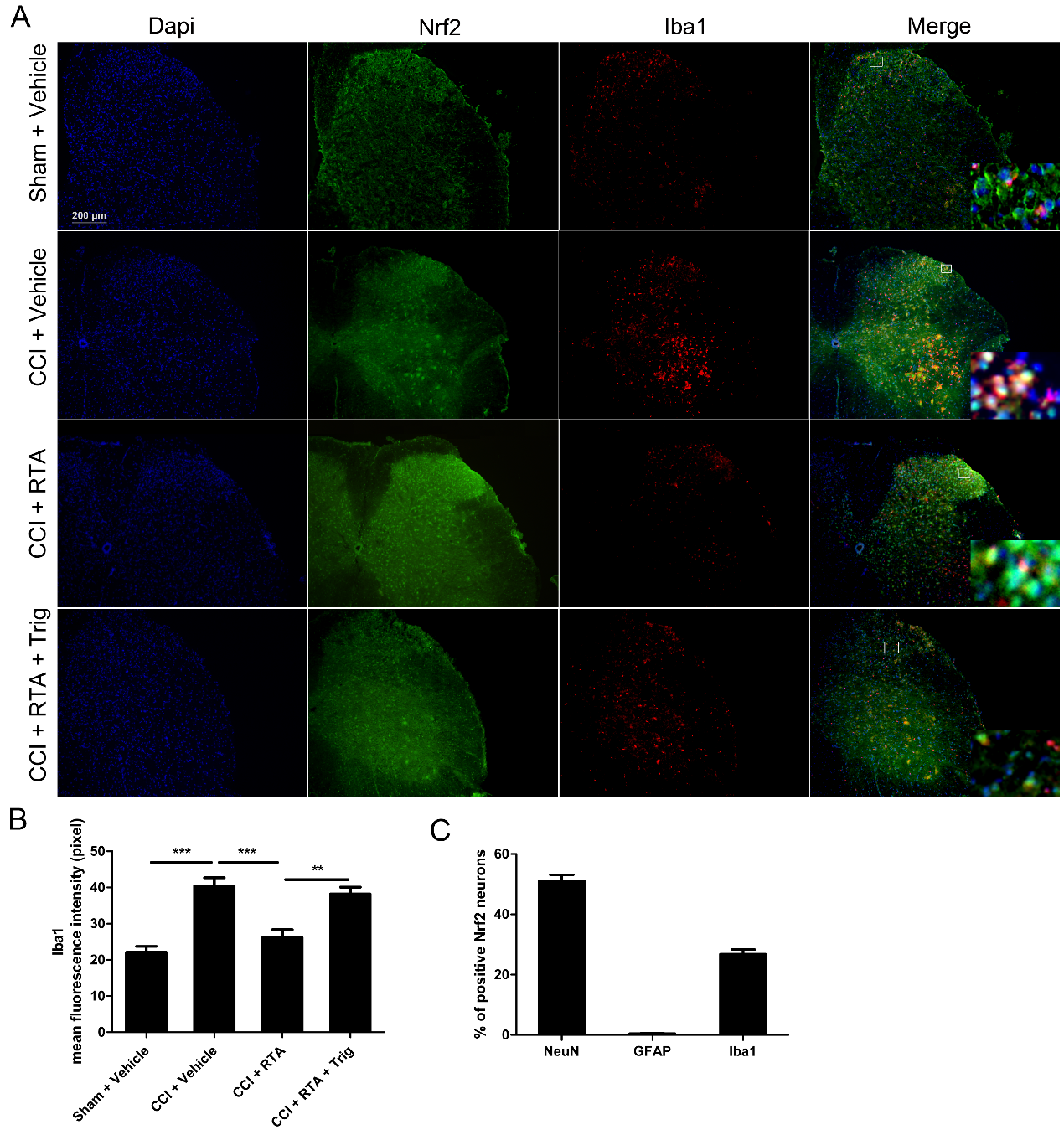

Supplement: Supplementary 2 — Supplementary Figure 2. (A) Representative images of double immunostaining showing colocalization of Iba1 (red) and Nrf2 (green) in the ipsilateral spinal cord dorsal horn (magnification: 100x and 1000x; scale bar = 200 μm and 10 μm). (B) The quantitative data of double immunofluorescence staining. (C) Double immunofluorescence staining showed that Nrf2 was mainly expressed in neurons but not in astrocyte cells in the spinal cord dorsal horn in CCI mice. Yellow represents colocalization. One-way ANOVA followed by Bonferroni analysis was used to test the differences among groups (∗∗P < 0.01, ∗∗∗P < 0.001 compared with the indicated group, n = 3 per group). [file 9577874.f2.docx]
